# Cytidinediphosphate diacylglycerol synthase—Mediated phosphatidic acid metabolism is crucial for early embryonic development of *Arabidopsis*

**DOI:** 10.1371/journal.pgen.1010320

**Published:** 2022-07-25

**Authors:** Xin-Qiao Du, Hong-Yan Yao, Pan Luo, Xing-Chun Tang, Hong-Wei Xue

**Affiliations:** 1 Shanghai Collaborative Innovation Center of Agri-Seeds, Joint Center for Single Cell Biology, School of Agriculture and Biology, Shanghai Jiao Tong University, Shanghai, China; 2 State Key Laboratory of Genetic Engineering, School of Life Sciences, Fudan University, Shanghai, China; 3 National Key Laboratory of Plant Molecular Genetics, CAS Center for Excellence in Molecular Plant Sciences, Chinese Academy of Sciences, Shanghai, China; 4 State Key Laboratory of Biocatalysis and Enzyme Engineering, College of Life and Science, Hubei University, Wuhan, China; Peking University, CHINA

## Abstract

Embryonic development is a key developmental event in plant sexual reproduction; however, regulatory networks of plant early embryonic development, particularly the effects and functional mechanisms of phospholipid molecules are still unknown due to the limitation of sample collection and analysis. We innovatively applied the microspore-derived *in vitro* embryogenesis of *Brassica napus* and revealed the dynamics of phospholipid molecules, especially phosphatidic acid (PA, an important second messenger that plays an important role in plant growth, development, and stress responses), at different embryonic developmental stages by using a lipidomics approach. Further analysis of *Arabidopsis* mutants deficiency of *CDS1* and *CDS2* (cytidinediphosphate diacylglycerol synthase, key protein in PA metabolism) revealed the delayed embryonic development from the proembryo stage, indicating the crucial effect of CDS and PA metabolism in early embryonic development. Decreased auxin level and disturbed polar localization of auxin efflux carrier PIN1 implicate that CDS-mediated PA metabolism may regulate early embryogenesis through modulating auxin transport and distribution. These results demonstrate the dynamics and importance of phospholipid molecules during embryo development, and provide informative clues to elucidate the regulatory network of embryogenesis.

## Author summary

As an important stage of plant life cycle, embryogenesis initiates with fertilization and terminates with embryo maturation, and plays important roles for seed development. In *Arabidopsis*, embryogenesis can be divided into three general phases during which distinct developmental and physiological events occur: proembryo stage that starts with asymmetric division of zygote, the transition from globular to heart stage, and late embryogenesis which is marked by organ expansion and maturation. Genetics studies have identified the genes and network that play important regulatory roles in embryogenesis, however, effects and functional mechanisms of phospholipids molecules, the major building blocks of cell membranes and signal transduction cascades in mammals and plants, in embryogenesis remain unknown. To overcome the limitation of sample collection, we innovatively applied the microspore-derived *in vitro* embryogenesis of *Brassica napus* and revealed the dynamics of phospholipid molecules, especially phosphatidic acid (PA) at different embryonic developmental stages by using a lipidomics approach. Further genetics analysis revealed the crucial effect of CDS (cytidinediphosphate diacylglycerol synthase, key protein in PA metabolism)—mediated PA metabolism in early embryonic development through modulating auxin transport and distribution, demonstrating the dynamics and importance of phospholipid molecules during embryo development.

## Introduction

Embryogenesis is an important stage of plant life cycle, which initiates with fertilization and terminates with the maturation of embryo. In *Arabidopsis*, embryo undergoes a highly ordered process of cell division and differentiation, during which the emerging tissues are specified and patterned [1]. Early embryogenesis is the critical developmental phase when the basic features of plant body are established: the apical-basal axis of polarity, distinct tissue layers, and both the root and shoot pole [2].

In *Arabidopsis*, pattern formation of embryo at early stages is regulated by various factors and profoundly controlled by signaling molecule auxin [3]. In early stages, auxin biosynthesis and polar transport interact to generate local auxin accumulation, which has a pivotal role in determining the embryo structure and size at beginning of seed development [3–7]. Polar subcellular localization of auxin efflux carriers, especially PIN proteins, ensures the directional movement of auxin [8]. After the first division of zygote, auxin is locally produced in suspensor cells [9], and PIN7 is polarly localized in these cells to mediate the directional auxin flow to proembryo [4]. At early globular stage, the onset of localized auxin biosynthesis in proembryo is required for PIN1 polarization in the inner proembryonic cells, resulting in a basal auxin response maximum and specification of the future root pole [1,4].

Phospholipids play an important role in plant growth and development. Phospholipids are composed of a class of lipids whose structure is based on a glycerol backbone, including phosphatidic acid (PA), phosphatidylcholine (PC), phosphatidylserine (PS), phosphatidylethanolamine (PE), phosphatidylinositol (PI) and phosphatidylglycerol (PG) [10]. Studies showed that PA serves as a lipid messenger to modulate plant growth, development, and responses to stresses, through regulating various signaling pathways particularly by binding target proteins [11], including root gravitropism through mediating auxin signaling [12], root hair patterning through regulating nuclear translocation of WEREWOLF transcription factor [13] or endoplasmic reticulum (ER) morphology by regulating the metabolism of other lipids [14].

PA is produced and metabolized through complex pathways, of which the cytidinediphosphate diacylglycerol synthase (CDS) catalyzes PA to cytidine diphosphate diacylglycerol (CDP-DAG), the important branch point intermediate of glycerolipid biosynthesis of prokaryotic and eukaryotic organisms [15]. There are five CDS isoforms in *Arabidopsis thaliana* [15,16], among them *CDS1*, *CDS2* and *CDS3* genes probably encode extraplastidial isoforms and have similar enzymatic properties [15,17]. *CDS1* and *CDS2* present redundant functions and knockout of both genes leads to suppressed growth, smaller cotyledons and shorter hypocotyls, and death ~2 weeks after germination. Chloroplasts of *cds1 cds2* double mutant were slightly smaller and accumulated much starch. Determination of glycerolipid compositions revealed that *cds1 cds2* double mutant accumulate more PA, which was eight-fold higher than WT seedlings, while PI and PG were decreased [17], indicating the importance of CDS activity for phospholipid metabolism and plant development.

Recent studies revealed the crucial role of PA in embryonic development of animals. Locally produced lysophosphatidic acid (LPA, an intermediate of PA pathway), stimulates PG production and regulates the oviductal functions, and further participates in fertilization and transport of gametes and embryo [18]. In addition, phospholipase D1 (PLD1)-produced PA activates mTORC1 kinase activity, which in turn promotes decidualization and the successful implantation of embryo [19]. However, whether and how phospholipid especially PA molecules participate in the regulation of early plant embryogenesis is still unknown.

Because of the relatively low sample amounts and technical difficulty, progress has been limited in understanding the developmental events of early embryogenesis. To overcome the limitation, the *Brassica* microspore system provides a useful alternative model for studying the early events of embryogenesis *in vitro* [20]. In this process, the microspore, upon exposure to heat treatment, can switch from their gametophytic development to suspensor-like structures, mimicking the zygotic embryos [21]. By using the *Brassica* microspore system and applying a lipidomics approach, we here showed the dynamics of phospholipid molecules during embryo development. Further genetics studies revealed that suppression of *CDS1* and *CDS2* leads to retarded embryo development, demonstrating the crucial roles of CDS and PA metabolism in early embryogenesis.

## Results

### Lipidomics analysis revealed the dynamics of phospholipids during embryonic development

**Fig 1:**
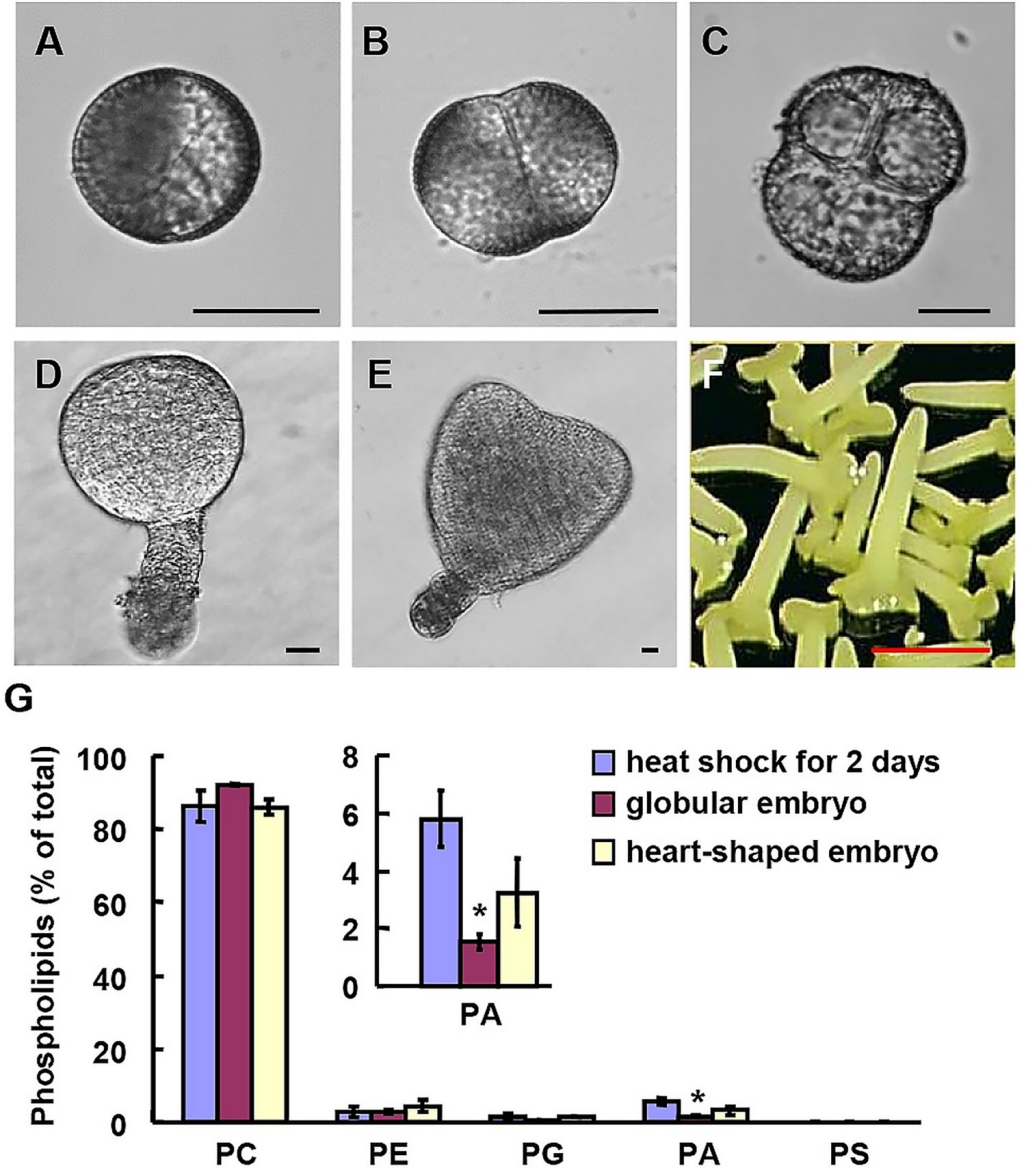
Dynamics of phospholipids at different stages during microspore embryogenesis of *Brassica napus*. (A-F) Microspore embryogenesis of *Brassica napus*, including vacuolated microspore (A), two-cells proembryo cultured for 48 h (B), four-cells proembryo cultured for 3 d (C), globular embryo with a suspensor cultured for 6 d (D), heart-shaped embryo with a suspensor cultured for 10 d (E), and cotyledonary embryos cultured for 28 d (F) after heat-shock treatment. Bars = 25 μm (A-E), or 1.5 mm (F). (G) Embryos at developmental stages A, D and E were collected and amounts of various phospholipids were measured by a lipidomics approach, which were shown as the percentage of total phospholipids. PC, phosphatidylcholine; PE, phosphatidylethanolamine; PG, phosphatidylglycerol; PA, phosphatidic acid; PS, phosphatidylserine. Experiments were performed with three technical replicates and data were mean ± SE. Statistical significance was determined by student’s *t* test (*, P<0.05).

To overcome the limitation of low sample amounts and technical challenge of preparation and analysis of phospholipids, which is particularly difficult using Arabidopsis, we applied the microspore-derived *in vitro* embryos of *Brassica napus* as an experimental system. After heat-shock treatment, the uni-nucleate microspores (Fig 1A) undergo the first symmetric division (Fig 1B), which initiates the microspore embryogenesis. Further mitotic divisions result in a multi-cellular structure (Fig 1C), globular embryo (Fig 1D) and heart-shaped embryo (Fig 1E), eventually developing the whole haploid plant (Fig 1F).

Microspore-derived *in vitro* embryos at different stages including globular and heart-shaped embryos were collected. These two stages are crucial for differentiation of embryonic organs and tissue-types, during which the regions along the longitudinal apical-basal axis differentiate from each other and the three primordial tissue layers of the embryo are specified. Further, by using microspores with heat-shock for 2 days as control, a lipidomic analysis was performed to quantify the content of various phospholipids using a ESI-MS/MS (S1 Table). Results showed that contents of PC, PE, PG and PS did not present obvious difference among the three examined stages (Fig 1G), while compared to microspore with heat-shock treatment for 2 days, PA content significantly decreased in globular embryo and increased slightly in heart-shaped embryo (Fig 1G), suggesting that PA may play a regulatory role in early embryogenesis. Detailed analyses indicated the decreased PA (32:0-, 32:1-, 32:2-, 34:2-, 34:3-, 34:4-, 36:2-, 36:3-, 36:4-, 36:5-, 38:3-, and 42:3-) and slightly increased PA (40:2-) species in globular embryo and heart-shaped embryo (S1 Fig).

Contents of phospholipid species 34:1-, 34:2-, 34:3-, 36:2-, 36:3–36:4-, 36:5-, 36:6-, 42:2-, 42:3- and 42:4- were higher than other species (S1–S5 Figs). Distinct species including 34:2-PC, 40:2-PE, 36:1-PG, 36:2-PG, 38:1-PS, 44:2-PS also presented obvious differences (S2–S5 Figs), suggesting these phospholipids species may involve in embryogenesis regulation as well with specific functions. Considering the significant changes of PA content and importance of PA in regulating growth and development, we focused on the role and functional mechanism of PA in regulating early embryonic development.

### *CDS1* and *CDS2* are transcribed during embryogenesis

To identify the candidate genes of PA metabolism that may regulate early embryonic development, expression patterns of genes involving in PA metabolism during *Arabidopsis* embryogenesis were investigated by publicly available data on eFP Browser (http://bar.utoronto.ca/efp/cgi-bin/efpWeb.cgi). Among the examined genes, *CDS1* and *CDS2*, two closely related genes encoding the cytidinediphosphate diacylglycerol synthase that catalyzes PA to cytidine diphosphate diacylglycerol (CDP-DAG), were expressed during embryogenesis, especially at the early developmental stages (S6 Fig).

**Fig 2:**
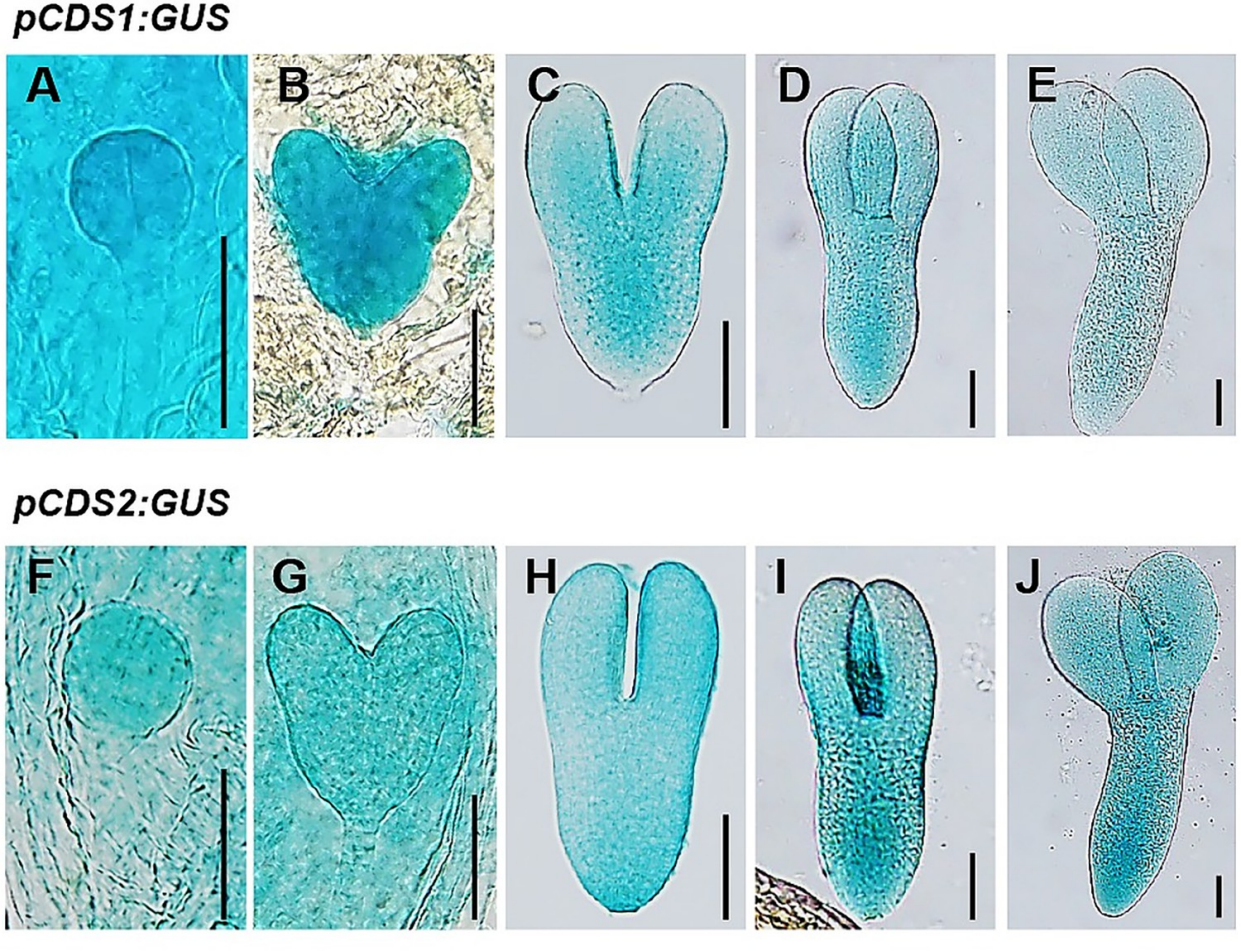
*CDS1* and *CDS2* genes are transcribed during *Arabidopsis* embryo development. Promoter-reporter gene (GUS) fusion study revealed that *CDS1* and *CDS2* were transcribed during *Arabidopsis* embryo development, including globular embryo (A, F), heart-shaped embryo (B, G), early torpedo embryo (C, H), late torpedo embryo (D, I) and cotyledon embryo (E, J). Representative images of three independent homozygous lines were shown. Bars = 50 μm.

Being consistent, promoter-reporter (GUS) fusion study by using promoter regions upstream of ATG of *CDS1* (2045-bp) or *CDS2* (1451-bp) showed that both *CDS1* and *CDS2* are transcribed at globular stage (Fig 2A and 2F). As the embryo/endosperm develops, *CDS1* is specially expressed in embryo and reached the maximum at heart-shaped stage (Fig 2B). From the torpedo stage onwards, *CDS1* transcription gradually became more pronounced in provascular cells (Fig 2C and 2D). Weak transcription of *CDS1* was detected in cotyledonary embryo (Fig 2E). Compared to *CDS1*, weak transcription of *CDS2* was also detected in suspensor and endosperm besides the embryo proper at the heart stage (Fig 2G), and increased significantly until late torpedo embryo (Fig 2I). At torpedo and cotyledonary stage, *CDS2* is mainly expressed in provascular cells (Fig 2I and 2J). Interestingly, expression level of *CDS2* in cotyledonary embryo was much higher than that of *CDS1* (Fig 2J). These suggested that CDS1 and CDS2 may function in the regulation of embryo development at early stages.

### Suppression of *CDS1* and *CDS2* resulted in delayed embryo development

**Fig 3:**
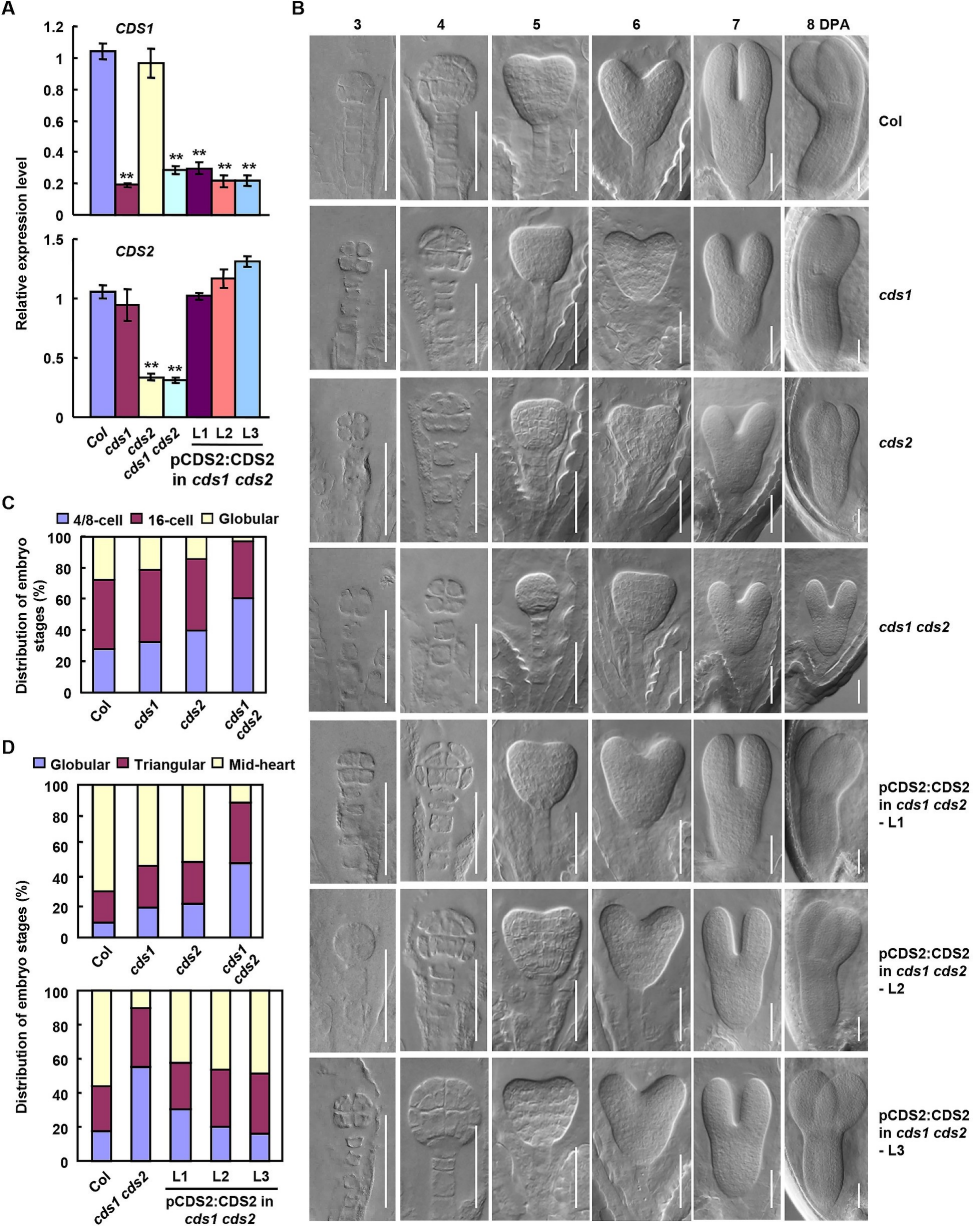
Suppressed expression of *CDS1* and *CDS2* resulted in the defective embryonic development. (A) RT-qPCR analysis of transcriptions of *CDS1* and *CDS2* genes in Col, various mutants, and *cds1 cds2* plants with complemented expression of *CDS2* driven by native promoter. *ACTIN2/8* was amplified and used as an internal reference to normalize the expressions of *CDS1* and *CDS2*. Experiments were biologically repeated for two times and data were mean ± SE. Statistical significance was determined by student’s *t* test (**, P<0.01). (B) Embryo development of Col, *cds1*, *cds2*, *cds1 cds2* mutants and *cds1 cds2* plants with complemented expression of *CDS2* driven by native promoter (pCDS2:CDS2 in *cds1 cds2*). Images of cleared seeds were taken by DIC microscopy at 3, 4, 5, 6, 7, 8 days post anthesis (DPA). Representative images were shown. Bars = 50 μm. (C) Quantitative analysis of embryos at different developmental stages (4/8-cell, 16-cell or globular) of siliques at 3 DPA of Col and various mutants. Experiments were biologically repeated and at least 89 seeds were observed. (D) Quantitative analysis of embryos at different developmental stages (globular, triangular or mid-heart) of siliques at 5 DPA of Col, various mutants, and *cds1 cds2* plants with complemented expression of *CDS2* driven by native promoter. Experiments were biologically repeated for two times. At least 172 or 136 seeds were observed for embryos of *cds* mutants (upper) or embryos of *cds1 cds2* mutants with complemented expression of *CDS2* driven by native promoter (bottom), respectively.

Significantly decreased PA level in globular embryo suggested the importance of PA metabolism. To detailed study the PA effects and whether altered PA lead to defective early embryonic development, T-DNA insertion mutants of *CDS1* and *CDS2* were identified (S7A and S7B Fig). RT-qPCR analysis confirmed the suppressed *CDS1* or *CDS2* transcription in *cds1*, *cds2* or *cds1 cds2* double mutants (Figs 3A and S7C).

Observation of embryo development during 3 to 8 days post anthesis (DPA) by differential interference contrast (DIC) microscopy of cleared seeds showed that compared with wild type (Col), *cds1* or *cds2* mutant presented a delayed, and *cds1 cds2* double mutant presented a severely delayed, embryonic development (Fig 3B). *cds1 cds2* embryos showed a slightly delayed proembryo development at 3 DPA, and more obvious delay during transition from globular to heart stage at 5 DPA (Fig 3B). When wild type embryos reached the mid-heart, late torpedo, and cotyledonary stage at 6 to 8 DPA, most of *cds1 cds2* embryos only reached the transition from globular to heart, mid-heart and early torpedo stage, respectively (Fig 3B).

Furthermore quantitative analysis of embryo development status showed that *cds1 cds2* embryos presented delayed development as early as the 16-cell stage. When most wild type embryos reached 16-cell stage at 3 DPA, *cds1 cds2* embryos were mainly at 4- or 8-cell stage (Fig 3C). More evident differences between wild type and *cds1 cds2* embryos were at the transition from globular to heart stage at 5 DPA (Fig 3D). In wild type, ~70% embryos reached heart stage while 52%, 50%, 11% of *cds1*, *cds2* and *cds1 cds2* respectively reached heart stage (Fig 3D, upper), the severely retarded embryogenesis indicated the importance of CDS and PA metabolism in early embryonic development. As expected, complemented *CDS2* expression (driven by its own native promoter, Fig 3A) rescued the defective embryonic development of *cds1 cds2* (Fig 3B and 3D bottom), confirming that CDS1 and CDS2 redundantly affected the early embryogenesis.

Previous study of *cds1 cds2* double mutants by crossing homozygous *cds1* (SALK_088268) and *cds2* (SALK_011704), of which the *CDS1* or *CDS2* transcripts were undetectable, showed that *cds1 cds2* knockout mutant was seedling lethal, failing to develop true leaves and died after germination [17]. In our study, the *cds1 cds2* double mutant grow normally as wild type except for the delayed embryonic development and shorter roots. The difference of growth abnormality may due to that *CDS1* and *CDS2* expression in *cds1 cds2* double mutant are down-regulated rather than knock-out in our study (S7 Fig).

### Decreased auxin level in *cds1 cds2* embryos

Studies in *Arabidopsis* showed that pattern formation of early embryo is profoundly controlled by auxin [3], which might directly cause cotyledon initiation in the apical margins of globular embryo [22]. To examine whether the delayed embryo development of *cds1 cds2* double mutant is due to the inappropriate level or distribution of auxin, a DR5::GFP marker (a synthetic promoter that responds to auxin) was introduced into *cds1 cds2* plants by cross.

**Fig 4:**
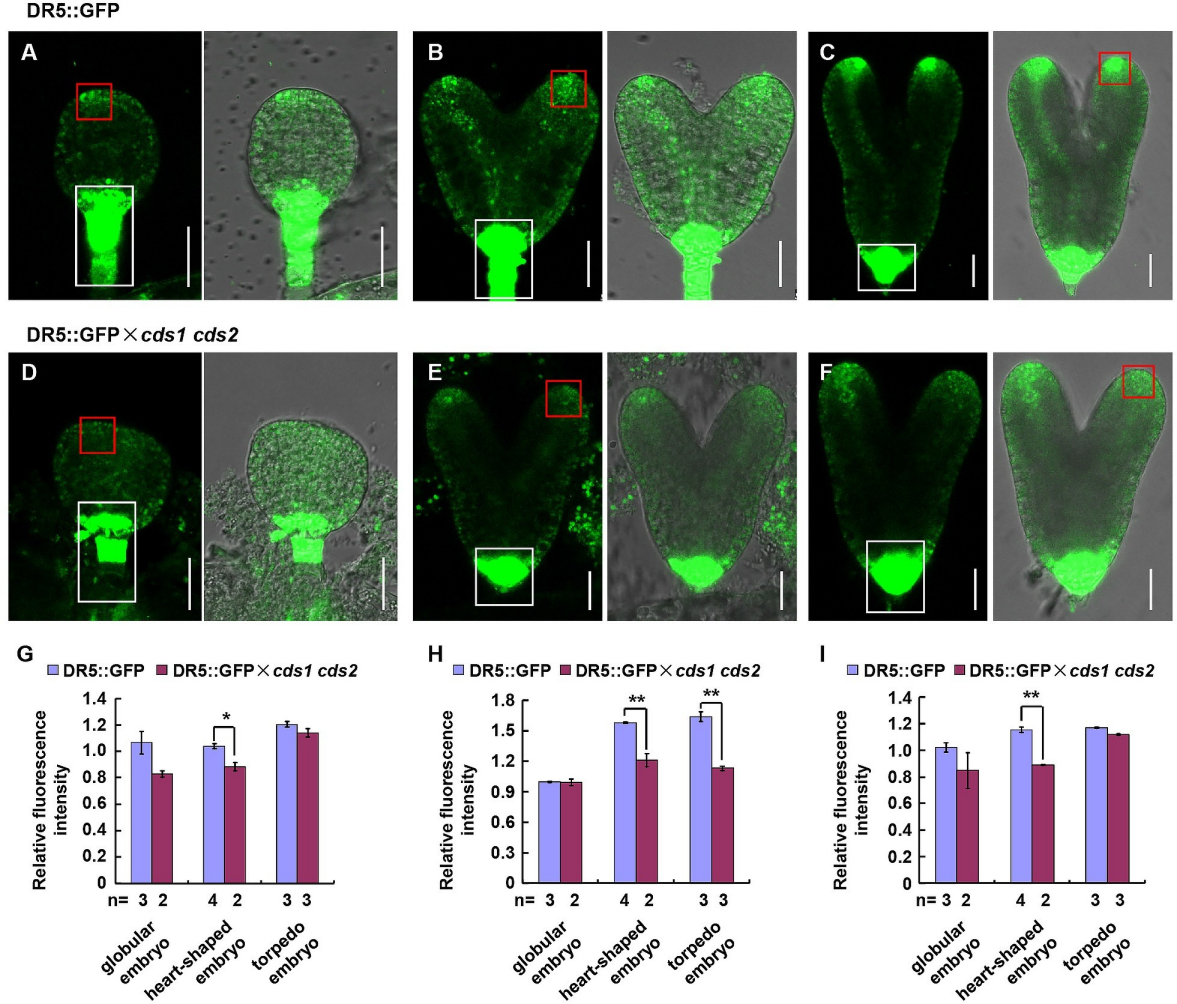
Suppressed expression of *CDS1* and *CDS2* resulted in altered auxin distribution during embryo development. (A-F) Auxin distribution in embryos at different developmental stages of Col and *cds1 cds2* mutant, including globular embryo (A, D), heart-shaped embryo (B, E) and torpedo embryo (C, F). *Arabidopsis* marker line DR5::GFP or offsprings of DR5::GFP cross with *cds1 cds2* (DR5::GFP × *cds1 cds2*) were analyzed. Representative images were shown. Bars = 25 μm. (G-I) Relative GFP fluorescence intensity of whole embryo (G), cotyledon primordia (H) and hypophysis and suspensor (I) at different developmental stages in DR5::GFP and DR5::GFP × *cds1 cds2*. Cotyledon primordia and hypophysis and suspensor are indicated by red or white boxes in A-F, respectively. Analyzed embryo numbers (n) from 2 independent experiments were indicated. Values were mean ± SE. Statistical significance was determined by student’s t test (*, p < 0.05; **, p < 0.01).

Observation of GFP fluorescence in various stages, especially early stages during embryonic development showed that in wild type embryos, an auxin maximum was detected in the uppermost suspensor cell of globular stage (hypophysis) (Fig 4A), and accumulated in hypophysis, cotyledon primordial tips, and prevascular cells from heart-stage to torpedo-stage (Fig 4B and 4C). Differently, a slightly decreased level of auxin was detected in suspensor of *cds1 cds2* embryos at globular stage (Fig 4D), and auxin in hypophysis and cotyledon primordia was significantly decreased at heart stage (Fig 4E). The polar distribution of auxin at cotyledons almost disappeared at torpedo stage, instead, auxin was evenly distributed throughout the entire protoderm (Fig 4F).

Further analysis by measuring the fluorescence intensity indeed revealed the reduced auxin level of whole embryo, particularly at heart-shaped stage (Fig 4G), and cotyledon primordia at heart-shaped and torpedo stages (Fig 4H), hypophysis and suspensor at heart-shaped stage (Fig 4I), under *CDS1* and *CDS2* deficiency, indicating that reduced auxin level and abnormal auxin distribution, particularly at heart-shaped stage, led to the retarded development of *cds1 cds2* embryos.

### Altered PIN1 localization in *cds1 cds2* embryos

Transport and polar distribution of auxin within plant organs is determined by activities of several distinct families of transporters, of which PIN-FORMED (PIN) members play critical roles in polar auxin transport during embryonic stages. Studies showed that PIN7 ensures the auxin transport from suspensor to pro-embryo until 16-cell stage. From 32-cell-stage, auxin is produced in the apical part of globular embryo and PIN1 plays a more important role to reverse the route of auxin, which is necessary for the formation of shoot meristem—cotyledon boundary region and initiation of cotyledon primordia [8,23]. Considering the delayed embryo development of *cds1 cds2* is more significant at transition from globular to heart stage (Fig 3B), during which cotyledon primordia begin to initiate, we thus examined the subcellular localization of PIN1 by crossing the marker line PIN1-YFP with *cds1 cds2* mutant.

**Fig 5:**
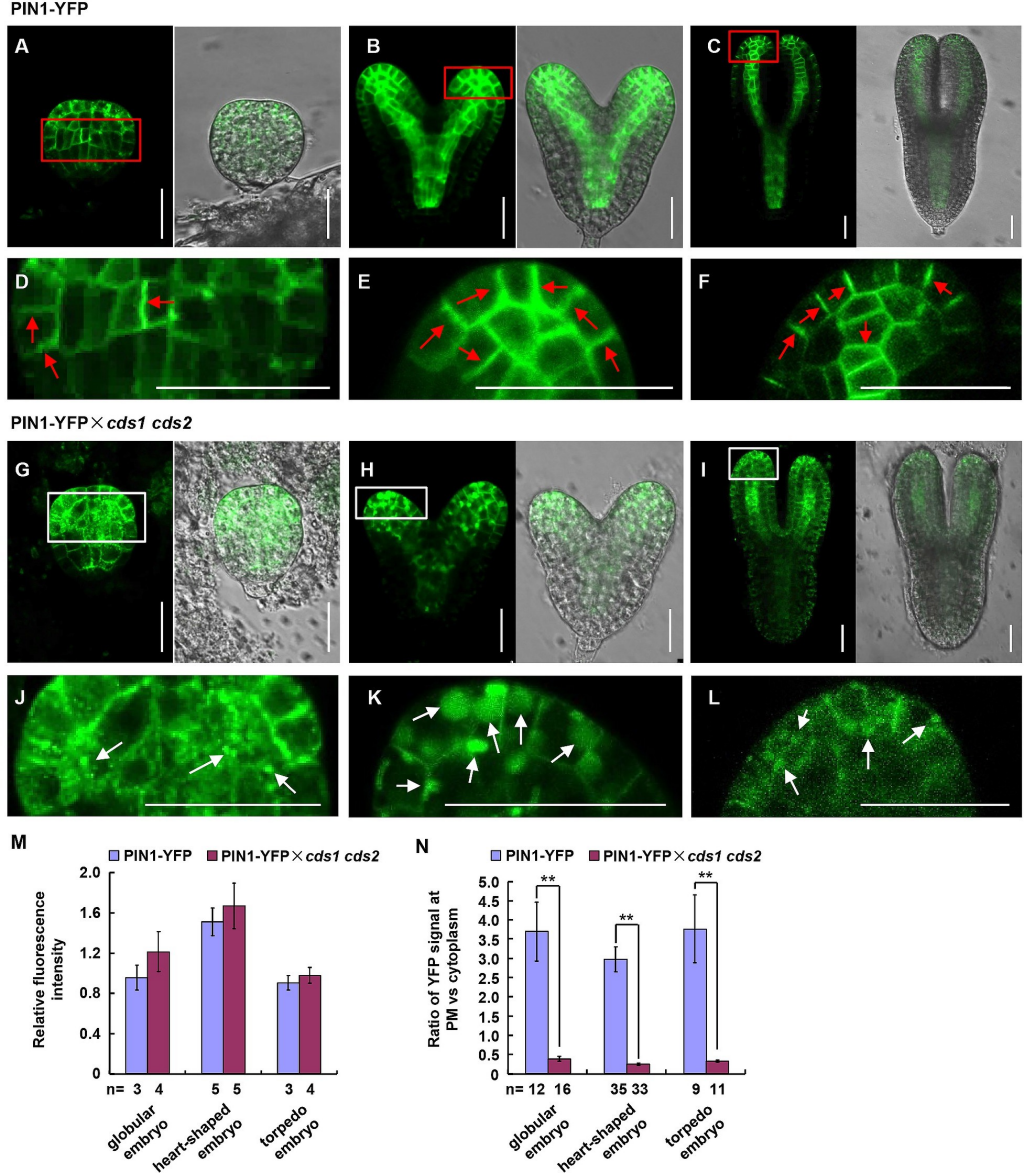
Disturbed polar localization of auxin efflux carrier PIN1 under suppressed expression of *CDS1* and *CDS2*. (A-L) Subcellular localization of auxin efflux carrier PIN1 in embryos at different developmental stages of Col and *cds1 cds2*, including globular embryo (A, G), heart-shaped embryo (B, H) and torpedo embryo (C, I). (D-F) Enlargement of A-C, respectively. Red arrows highlighted the polar localization of PIN1 at plasma membrane. (J-L) Enlargement of G-I, respectively. White arrows highlighted the dot structure of PIN1 in cytoplasm. *Arabidopsis* line harboring PIN1-YFP fusion protein or offsprings of PIN1-YFP cross with *cds1 cds2* (PIN1-YFP × *cds1 cds2*) were analyzed. Observations were performed with 2 independent lines and representative images were shown. Bars = 25 μm. (M-N) Relative YFP fluorescence intensity (M) and ratio of YFP signal intensity at PM to cytoplasm (N) at different embryo developmental stages in PIN1-YFP and PIN1-YFP × *cds1 cds2*. Numbers of analyzed embryos or cells (n) from 2 independent experiments were indicated. Values were mean ± SE. Statistical significance was determined by student’s t test (**, p < 0.01).

In wild type, PIN1 localized at plasma membranes of globular embryos (Fig 5A and 5D), and mainly in vascular precursor cells and epidermal cell layer of cotyledon primordia from heart-shaped to torpedo stage (Fig 5B, 5C, 5E and 5F). In addition, PIN1 localized predominantly to the basal side of plasma membrane. However, the polar localization of PIN1 is significantly defected in *cds1 cds2* embryos. At globular stage, PIN1 showed dot-like structure in cytoplasm and nonpolar distribution in cells of embryo proper (Fig 5G and 5J). At heart stage, PIN1 was confined to cytoplasm and hardly detectable at plasma membrane, and the PIN1 protein level was dramatically reduced in vascular precursor cells (Fig 5H and 5K). In torpedo-shaped *cds1 cds2* embryos, PIN1 still showed diffused distribution, and PIN1 protein level was increased in epidermal cell of embryos and reduced in vascular precursor cells (Fig 5I and 5L).

Consistently, analysis of PIN1 localization by measuring fluorescence intensity showed that PIN1 level was unaltered at different developmental stages of *cds1 cds2* embryos (Fig 5M), however, the polar localization is significantly defected, i.e. much reduced accumulation at plasma membranes at various stages (Fig 5N). These results indicated that CDS1/CDS2-mediated PA metabolism regulated early embryonic development by influencing the polar transport and distribution of auxin.

## Discussion

### Dynamic phospholipids during embryonic development

Accurate and comprehensive detection and identification of phospholipids, as well as the molecular species of different phospholipids, is crucial for studying their functions in plant growth, development, and responses to environmental stresses. Mass spectrometry and lipidomic analyses by direct-infusion electrospray ionization triple quadrupole mass spectrometry (MS) with electrospray ionization (ESI) improved mass scan speed and sensitivity, which makes it possible to identify molecular species of phospholipids and significantly helps to study the *in vivo* function of genes involving in lipid metabolism [24–26]. However, due to the difficulty of collecting plant embryos that are surrounded by endosperm and seed coat, it is hard to determine the contents and dynamics of phospholipids and distinct species during embryogenesis, particularly the early stages, of *Arabidopsis*.

By using microspore-derived *in vitro* embryos of *B*. *napus* as an experimental system, we were able to conduct the lipidomic analyses and as first time, revealed the phospholipids dynamics in early embryos (Fig 1), providing informative cues for functional studies of lipid metabolism during early embryogenesis. Lipidomic analyses also revealed the possible differential roles of distinct phospholipids molecular species during embryogenesis. Specific PA species presented obvious differences at different embryonic stages, so do other phospholipids, suggesting that molecular species of different phospholipids may play distinct roles during early embryogenesis.

With the increase of unsaturation, temperature of lipid molecule transition from gel phase to liquid crystal phase decreases, and the fluidity of cell membrane increases [27], which affects the membrane trafficking and hence various signaling pathways. In addition, phospholipids with different saturations have differential binding abilities to target proteins, for example, PC species with linolenic acid (36:5 and 36:6) bind poorly to FLOWERING LOCUS T (FT) by contrast to less unsaturated PC species [28]. In our study, changed phospholipids during early embryonic development mainly include species containing less unsaturated fatty acids such as 34:2-, 34:3-, 36:4-, 42:3- and 40:2-PA, 34:2-PC, 40:2-PE, 36:1-PG, 36:2-PG, 38:1-PS, 44:2-PS, while content of species containing polyunsaturated (5 and 6 double bonds) fatty acid had no significant difference except for 36:5-PA (S1–S5 Figs). We hypothesize that phospholipids with low saturation are key regulators for early embryonic development, possibly through altering membrane fluidity and binding ability to target proteins.

### CDS-mediated PA metabolism regulates early embryonic development

PA plays key roles during plant development and stress responses [11], however, its function in embryonic development, particularly the early stage, is still unknown. In this study, we revealed that PA metabolism is crucial in regulating early embryonic development in plants. PA production and metabolism are mediated by a variety of enzymes including CDS and we showed that CDS1/CDS2 deficiency leads to delayed embryonic development, demonstrating the importance of CDS1/CDS2-mediated PA metabolism in early embryonic development.

CDS1 and CDS2 proteins are localized on ER membrane [17], which is the main organelle to produce phospholipids and provide lipids being transported to other organelles. Actually, galactolipids which are the major membrane lipids of chloroplasts and other phospholipids also showed pronounced changes in *cds1 cds2* mutant, except for significantly elevated PA levels [17]. Organelle formation plays important roles to ensure the normal embryo development. Therefore, CDS1/CDS2-mediated PA metabolism may also participate in the maintenance of lipid homeostasis and organelle function, and further regulates the early embryonic development.

Some other enzymes including PLD and PLC/DGK are also involved in PA metabolism, however, function of them in early embryogenesis has not been reported yet. Our studies suggested the distinct role of CDS-derived PA metabolism in regulating early embryonic development. In fact, PLD and PLC/DGK pathway generated PA species with different molecular properties due to differences in hydrocarbon chains (length and unsaturated bond number), thus regulating different biological processes [29,30]. We hypothesized that specific PA molecular species regulated by CDS may play a role in early embryonic development. *Arabidopsis* genome is predicted to encode 12 PLDs, 9 PLCs, 7 DGKs, and CDS isoforms are encoded by five genes, and only CDS1, CDS2 and CDS3 are extraplastidial ones [17], making it relatively easier to identify the function in early embryogenesis with less functional redundancy compared to PLDs, PLCs or DGKs.

Auxin has been demonstrated as a key regulator in early embryogenesis, including establishment of the apical-basal axis, hypophysis specification, root pole formation, and initiation of cotyledon primordia [2]. However, little is known about whether PA involves in early embryogenesis through regulating auxin distribution or signaling. Our results indicated the delayed embryo development of *cds1 cds2* mutant may be caused by altered PIN1 localization at plasma membrane (Fig 5), which ultimately disrupted auxin distribution in embryos (Fig 4). The change of PIN1 polarity in *cds1 cds2* mutant may be caused by direct binding of PA to PIN1 protein or other regulatory proteins, which may affect the phosphorylation, vesicle trafficking, and thus the polar localization of PIN1 at plasma membrane. Indeed, PA interacts with PP2AA1, subunit of Protein Phosphatase 2A (PP2A), and PINOID (PID) kinase to alter the phosphorylation of PIN1 and auxin polar transport [31,32], suggesting that PA metabolism and hormone coordinately regulate the early embryonic development of plants.

## Materials and methods

### Microspore isolation and culture

Microspores were collected from buds (2.8–3.2 mm in length) of *Brassica napus*. Generation of embryos using microspores was performed according to Tang et al. (2013). Briefly, microspores were isolated from anther by crushing buds in B5 medium [33] and filtering the suspension through a 30 μm mesh. Then the microspore suspension was centrifuged for three times at 100 g for 5 min each, the supernatant was removed, and microspores were resuspended in NLN-13 medium [34]. Microspore density was adjusted to 40,000 microspore/mL with NLN13 medium. An aliquots of 1 mL of microspore suspension were plated in 3 cm Petri dishes for culture in darkness with a heat-shock of 2 day at 32°C. Afterward, cultures were kept continuously at 25°C in darkness until the initiation of embryos. Each dish produced ~ 2000–3000 globular embryos or 1500–2000 heart-shaped embryos. Embryo materials of 4–5 dishes were collected for each sample.

### Plant material and growth conditions

The Arabidopsis (*Arabidopsis thaliana*) transfer DNA insertion lines (Columbia ecotype, Col-0) *cds1* (SALK_001496) and *cds2* (SALK_106246) were obtained from Nottingham Arabidopsis Stock Centre. *CDS2* genomic sequence was cloned into pCAMBIA1300 vector driven by its native promoter, and this construct was transformed into *cds1 cds2* double mutant to obtain the complementation lines (pCDS2:CDS2 in *cds1 cds2*). *CDS1* and *CDS2* promoter fragments were cloned into pCAMBIA1300 vector fused with the *GUS* reporter gene and were transformed into wild type plants to generate *pCDS1*:*GUS* or *pCDS2*:*GUS* plants, respectively.

*A*. *thaliana* seeds were surface sterilized using NaClO, plated on half-strength Murashige and Skoog (MS) medium (pH 5.8) containing 0.8% agar and 2% sucrose, stored for 2–4 days at 4°C, and grown vertically at 22°C under 16-h light/8-h dark condition. For seed harvest, plants were grown in a potting soil mixture (rich soil/vermiculite = 2:1, v/v) in growth chambers (22°C, illumination at 120 μmol m^-2^ s^-1^ for a 16-h daily light period; relative humidity ~70%).

Sequences of used primers were listed in S2 Table.

### Lipid extraction

Total lipids of different developmental stages including vacuolated microspore after a heat shock for two days, globular embryos and heart-shaped embryos were extracted according to a previous description [26] with few modifications. Harvested tissues were immediately transferred to 1 mL preheated isopropanol (75°C with 0.01% butylated hydroxytoluene). After incubating for 15 min, 0.5 mL chloroform and 0.2 mL water were added. Tubes were shaken for 1 h at room temperature, followed by removal of extract to a new glass tube. Tissues were re-extracted with 0.7 mL chloroform: methanol (2:1, v:v) five times with 30 min of agitation each time. Combined extracts were washed once with 0.33 mL KCl (1 mol/L) and once with 0.66 mL water. The solvent was evaporated under nitrogen and lipid extract was dissolved in 1 mL chloroform. The resultant solvent extracts were used for mass spectrometric analysis. Remaining tissues after lipid extraction were heated at 105°C, and dry weights were measured.

### Mass spectrometric lipid analysis

Lipid extract in chloroform was combined with solvents (chloroform: methanol: 300 mM ammonium acetate; 300:665:35; v:v:v) to final concentration 6 μg/μL of samples, and 50 μL was used for analysis. Internal standards were obtained from Avanti Polar Lipids and added in each sample, making final concentrations in sample as 16.34 μmol/L 17:0–14:1-PA, 0.167 μmol/L 17:0–20:4-PC, 0.205 μmol/L 17:0–20:4-PE, 0.171 μmol/L 21:0–22:6-PG, and 0.220 μmol/L 17:0–14:1-PS [35].

Samples were analyzed by flow injection using a Shimadzu CBM-20A Lite HPLC system (Kyoto, Japan) with a solvent mixture of 10 mM ammonium acetate in methanol. A flow gradient was performed as follows in sequence: 100 μL/min, 5 s; 30 μL/min, 1.7 min; 150 μL/min, 1 min. Mass spectrometric analysis was performed on an AB Sciex Qtrap 5500 triple quadrupole mass spectrometer (Framingham, MA, USA) equipped with a Turbo V electrospray ion source with the following parameters: ion spray voltage, 5500 V; entrance potential, 10 V; source temperature, 300°C; curtain gas, 25 (nitrogen); two ion source gases, 30; collision gas, medium. PC species were detected in positive ion mode by precursor scanning, and PA, PE, PG and PS species were determined in positive ion mode by neutral loss scanning, according to the previous description [26]. Mass spectra were processed and analyzed by LipidView Software (AB Sciex, version 1.1) for identification and quantification of phospholipids as previously described [36]. Lipid levels for each sample were calculated relative to internal standards and then normalized to total amount of all lipids measured.

### Promoter-GUS histochemical staining

GUS staining was performed according to Robert et al. (2015). Seeds were dissected from siliques and incubated under vacuum for 10 min in 90% acetone. Subsequently samples were washed twice under vacuum for 10 min with 0.5 M phosphate buffer [Na_2_HPO_4_/NaH_2_PO_4_ (615/385), pH 7.0], and then transferred to GUS staining solution [Triton X-100 (0.5% v/v); EDTA (1 mM); K_3_Fe(CN)_6_ (0.5 mM); K_4_Fe(CN)_6_ (0.5 mM); X-Glu (1 mM) dissolved in DMFO (0.5% v/v); phosphate buffer (0.5 M); pH 7.0]. After vacuum infiltration for 1 h, samples were incubated at 37°C. The staining reaction was terminated by adding 0.5 M phosphate buffer and then infiltrating under vacuum for 10 min. Embryos were transferred to slides with 10% glycerol, and imaged with a differential interference contrast (DIC)-fluorescence microscope (Olympus).

### Embryo phenotyping

For all experiments, siliques were freshly harvested. After being dissected from siliques, seeds were cleared at indicated stages in chloral hydrate solution (chloral hydrate: H_2_O: glycerol, 8:3:1, w/v/v). Images were taken under a microscope with DIC optics. For quantitative analysis of embryos at 3 and 5 DPA, all embryos from harvested siliques were calculated.

### Quantitative real-time RT-qPCR analysis

Total RNAs were extracted from 8-day-old seedlings using TRIzolR reagent (Invitrogen), incubated with DNAase (TaKaRa) and reverse transcribed (Toyobo). qPCR was carried out in a Bio-Rad CFX Connect Real-Time System using the SYBR Green qPCR kit (Toyobo). Amplification reactions were performed in a total volume of 20 μL, which contained 2 μL forward and reverse primers (1 μM), 1 μL cDNA, 10 μL SYBR Green premix, 7 μL distilled, deionized water. The procedure of qPCR was conducted as follows: 95°C for 10 min; 40 cycles of 95°C for 15 s and 60°C for 1 min. Relative expression levels were normalized to *ACTIN2/8*.

Sequences of used primers were listed in S2 Table.

### Confocal imaging

Seeds of GFP/YFP tagged lines of various developmental stages were collected into 10% (v/v) glycerol from developing inflorescences of plants. Embryos were pressed out from seeds by using a mild pressure applied to glass cover. Afterwards, distribution of GFP/YFP signals at various stages of embryogenesis for auxin sensor DR5::GFP and auxin transporter PIN1-YFP were analyzed using Leica TCS SPS-II confocal laser scanning microscopy. The manufacturer’s default settings were used for imaging proteins tagged with GFP (excitation, 488 nm; emission, 495–545 nm), and YFP (excitation, 514 nm; emission, 524–580 nm). Fluorescence intensities were quantified using ImageJ.

## Supporting information

S1 Fig Phosphatidic acid molecular species composition during microspore embryogenesis of *Brassica napus*. Lipids of different stages during microspore embryogenesis i.e. uni-nucleate microspores after heat-shock treatment for 2 days, globular embryo and heart-shaped embryo, were extracted and phosphatidic acid (PA) molecular species composition was analyzed by a lipidomic approach. Experiments were performed with three technical replicates and data were shown as the percentage of each PA type in total phospholipids (mean ± SE). Statistical significance was determined by student’s *t* test (*, p < 0.05; **, p <0.01).
(TIF)

S2 Fig Phosphatidylcholine molecular species composition during microspore embryogenesis of *Brassica napus*. Lipids of different stages during microspore embryogenesis i.e. the uni-nucleate microspores after heat-shock treatment for 2 days, globular embryo and heart-shaped embryo, were extracted and phosphatidylcholine (PC) molecular species composition was analyzed by a lipidomic approach. Experiments were performed with three technical replicates and data were shown as the percentage of each PC type in total phospholipids (mean ± SE). Statistical significance was determined by student’s *t* test (*, p < 0.05; **, p <0.01).
(TIF)

S3 Fig Phosphatidylethanolamine molecular species composition during microspore embryogenesis of *Brassica napus*. Lipids of different stages during microspore embryogenesis i.e. the uni-nucleate microspores after heat-shock treatment for 2 days, globular embryo and heart-shaped embryo, were extracted and phosphatidylethanolamine (PE) molecular species composition was analyzed by a lipidomic approach. Experiments were performed with three technical replicates and data were shown as the percentage of each PE type in total phospholipids (mean ± SE). Statistical significance was determined by student’s *t* test (*, p < 0.05; **, p <0.01).
(TIF)

S4 Fig Phosphatidylglycerol molecular species composition during microspore embryogenesis of *Brassica napus*. Lipids of different stages during microspore embryogenesis i.e. the uni-nucleate microspores after heat-shock treatment for 2 days, globular embryo and heart-shaped embryo, were extracted and phosphatidylglycerol (PG) molecular species composition was analyzed by a lipidomic approach. Experiments were performed with three technical replicates and data were shown as the percentage of each PG type in total phospholipids (mean ± SE). Statistical significance was determined by student’s *t* test (*, p < 0.05; **, p < 0.01).
(TIF)

S5 Fig Phosphatidylserine molecular species composition during microspore embryogenesis of *Brassica napus*. Lipids of different stages during microspore embryogenesis i.e. the uni-nucleate microspores after heat-shock treatment for 2 days, globular embryo and heart-shaped embryo, were extracted and phosphatidylserine (PS) molecular species composition was analyzed by a lipidomic approach. Experiments were performed with three technical replicates and data were shown as the percentage of each PS type in total phospholipids (mean ± SE). Statistical significance was determined by student’s *t* test (*, p < 0.05; **, p < 0.01).
(TIF)

S6 Fig Expression patterns of *CDS1* and *CDS2* during embryo development. Expression of *CDS1* and *CDS2* during *Arabidopsis* embryo development was analyzed on eFP Browser (http://bar.utoronto.ca/efp/cgi-bin/efpWeb.cgi). Different embryonic stages from 8-cell/16-cell to mature embryo were analyzed. Absolute expression levels of gene in different tissues were shown (right, the highest expression was indicated in red).
(TIF)

S7 Fig Identification of *Arabidopsis cds1* and *cds2* T-DNA insertional mutants. (A) Schematic representation of *CDS1* and *CDS2* genes and positions of T-DNA insertions for *cds1* and *cds2*. Introns, exons, and non-coding regions are indicated by lines, black, or blank boxes respectively. Positions of primers used were indicated. (B) Identification of homozygous *cds1*, *cds2* and *cds1 cds2* mutants by PCR. Homozygous lines have a single amplified DNA fragment when using LBb1.3/*cds1*-RP or LBb1.3/*cds2*-RP primers. (C) RT-qPCR analysis confirmed the significantly reduced expression of *CDS1* and *CDS2* genes in *cds1* and *cds2* mutants, respectively. *ACTIN2/8* was amplified and used as an internal reference to normalize the expression of *CDS1* and *CDS2*, and relative expression was calculated by defining the expression in Col as 1.0. Experiments were biologically repeated for three times and values were mean ± SE. Statistical significance was determined by student’s *t* test (*, p < 0.05; **, p < 0.01).
(TIF)

S1 Table Quantification of various phospholipids in uni-nucleate microspores after heat-shock treatment for 2 days, globular embryo and heart-shaped embryo by lipidomic analysis using a ESI-MS/MS. Experiments were performed with three technical replicates and data were shown as the percentage of each type of phospholipid molecule in total phospholipids.
(XLSX)

S2 Table Sequences of primers used in this study. Added restriction enzyme site was underlined.
(DOCX)
