# Supplementary figures and images for: Cytidinediphosphate diacylglycerol synthase—Mediated phosphatidic acid metabolism is crucial for early embryonic development of Arabidopsis
Source: PLoS Genet. 2022 Jul 25;18(7):e1010320. doi: 10.1371/journal.pgen.1010320 (PMC9352201; doi:10.1371/journal.pgen.1010320)

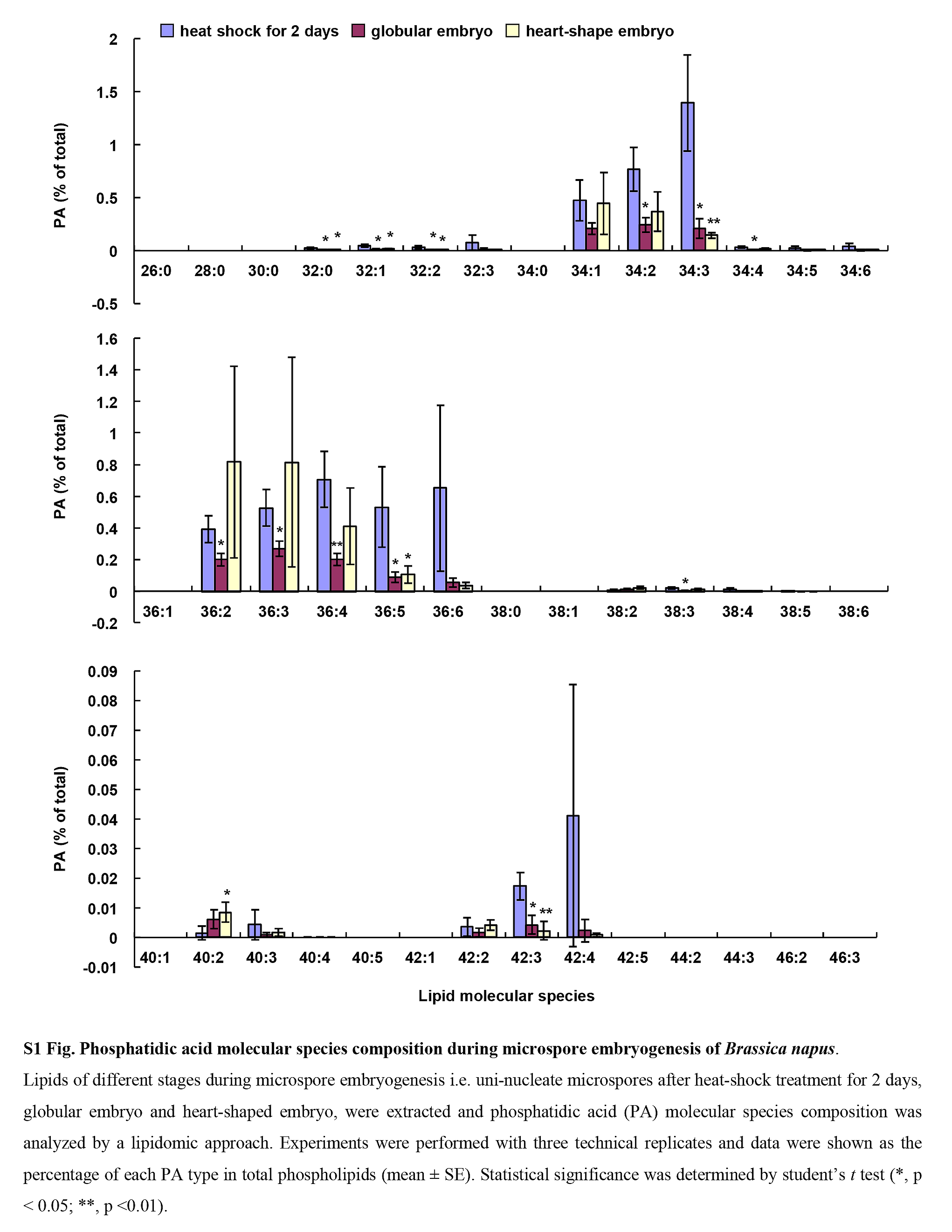

Supplement: S1 Fig — Lipids of different stages during microspore embryogenesis i.e. uni-nucleate microspores after heat-shock treatment for 2 days, globular embryo and heart-shaped embryo, were extracted and phosphatidic acid (PA) molecular species composition was analyzed by a lipidomic approach. Experiments were performed with three technical replicates and data were shown as the percentage of each PA type in total phospholipids (mean ± SE). Statistical significance was determined by student’s t test (*, p < 0.05; **, p <0.01). (TIF) [file pgen.1010320.s001.tif]

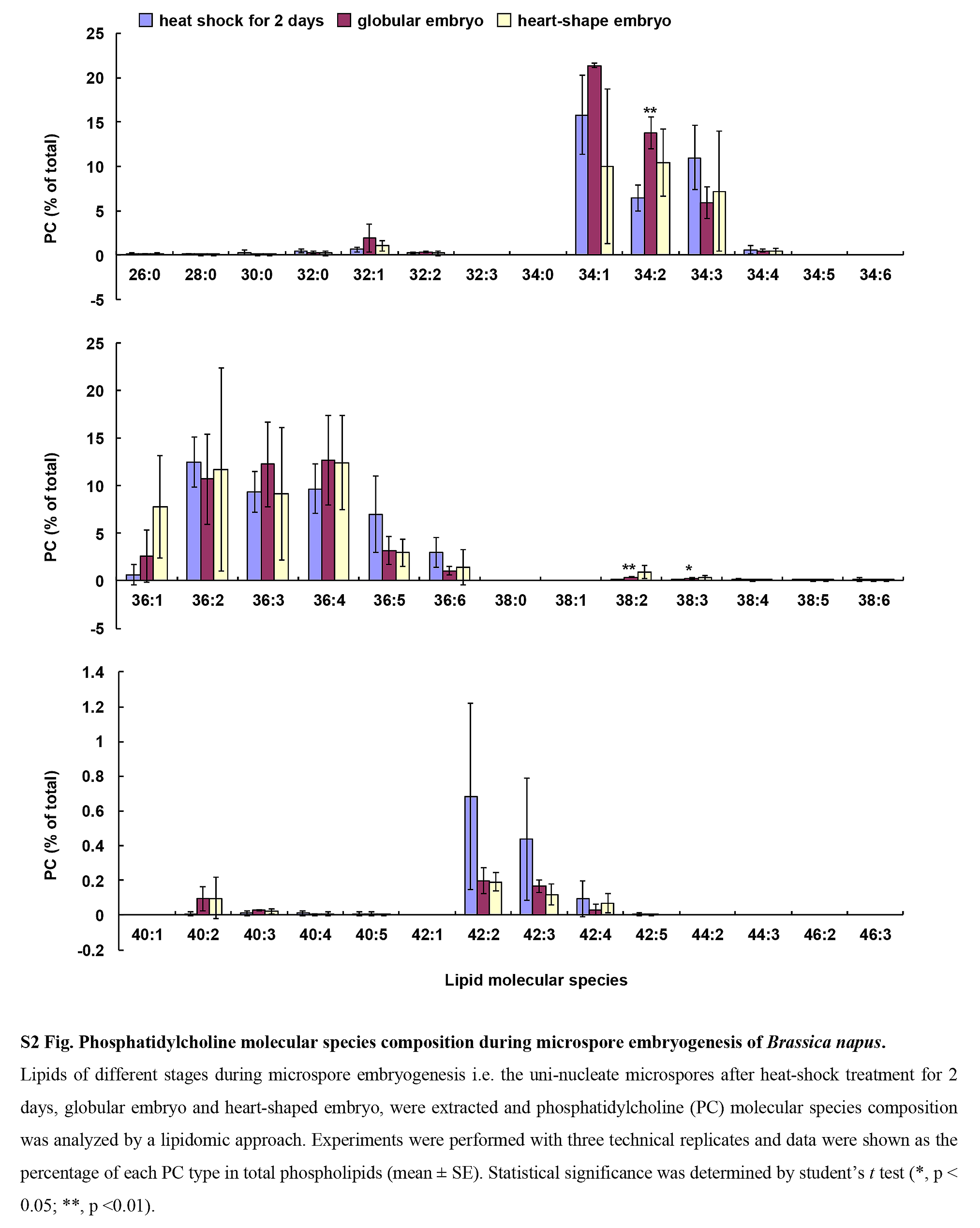

Supplement: S2 Fig — Lipids of different stages during microspore embryogenesis i.e. the uni-nucleate microspores after heat-shock treatment for 2 days, globular embryo and heart-shaped embryo, were extracted and phosphatidylcholine (PC) molecular species composition was analyzed by a lipidomic approach. Experiments were performed with three technical replicates and data were shown as the percentage of each PC type in total phospholipids (mean ± SE). Statistical significance was determined by student’s t test (*, p < 0.05; **, p <0.01). (TIF) [file pgen.1010320.s002.tif]

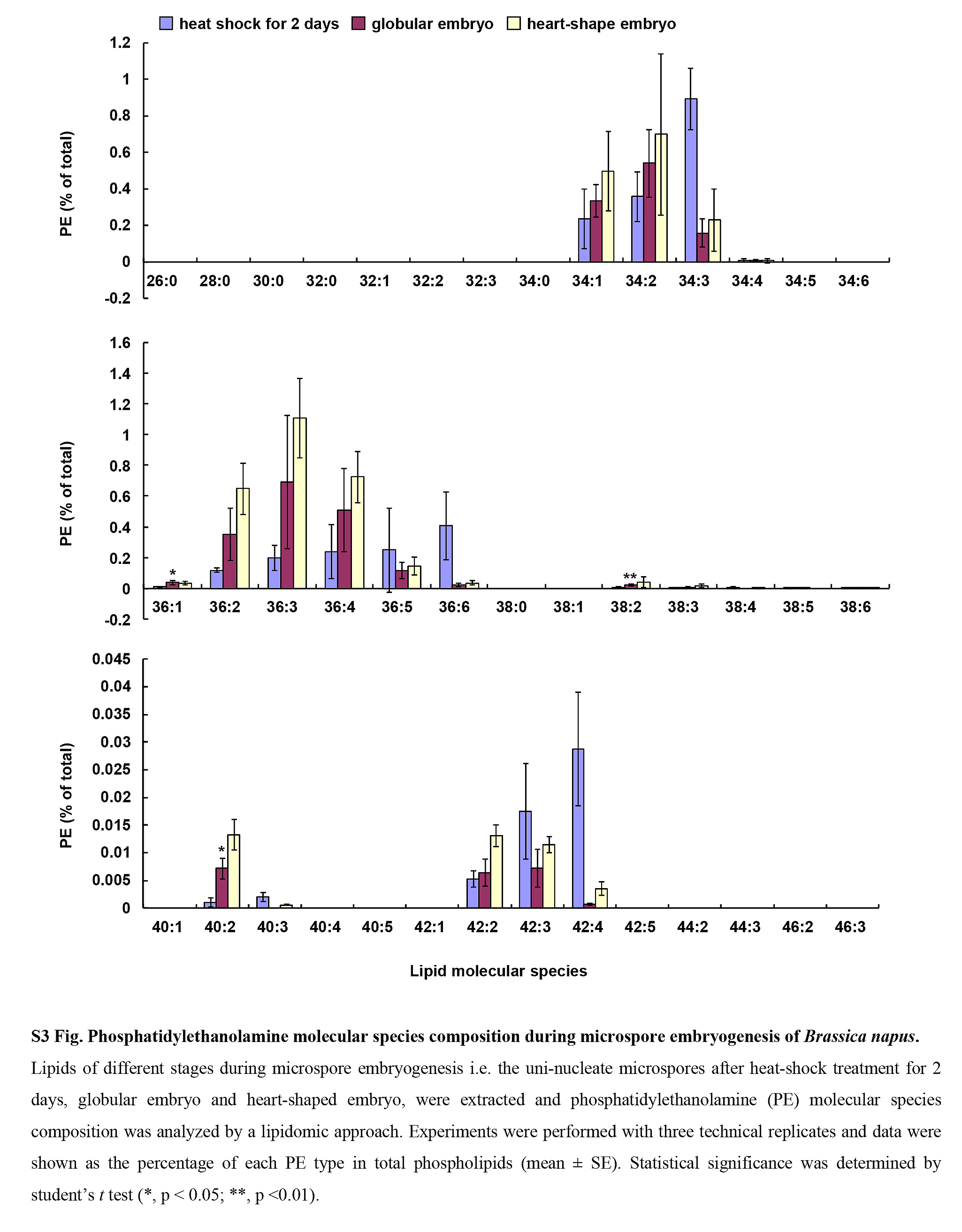

Supplement: S3 Fig — Lipids of different stages during microspore embryogenesis i.e. the uni-nucleate microspores after heat-shock treatment for 2 days, globular embryo and heart-shaped embryo, were extracted and phosphatidylethanolamine (PE) molecular species composition was analyzed by a lipidomic approach. Experiments were performed with three technical replicates and data were shown as the percentage of each PE type in total phospholipids (mean ± SE). Statistical significance was determined by student’s t test (*, p < 0.05; **, p <0.01). (TIF) [file pgen.1010320.s003.tif]

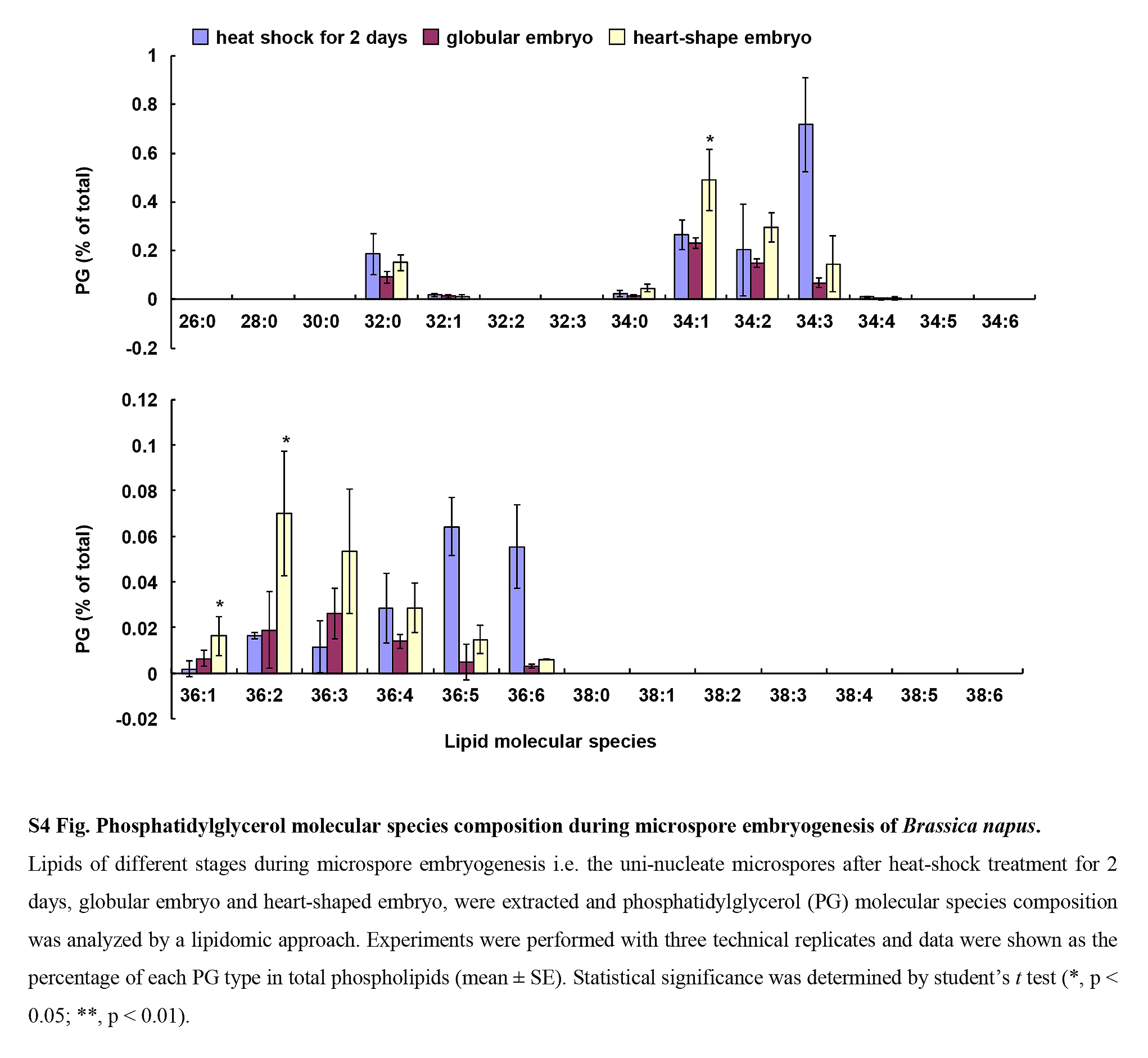

Supplement: S4 Fig — Lipids of different stages during microspore embryogenesis i.e. the uni-nucleate microspores after heat-shock treatment for 2 days, globular embryo and heart-shaped embryo, were extracted and phosphatidylglycerol (PG) molecular species composition was analyzed by a lipidomic approach. Experiments were performed with three technical replicates and data were shown as the percentage of each PG type in total phospholipids (mean ± SE). Statistical significance was determined by student’s t test (*, p < 0.05; **, p < 0.01). (TIF) [file pgen.1010320.s004.tif]

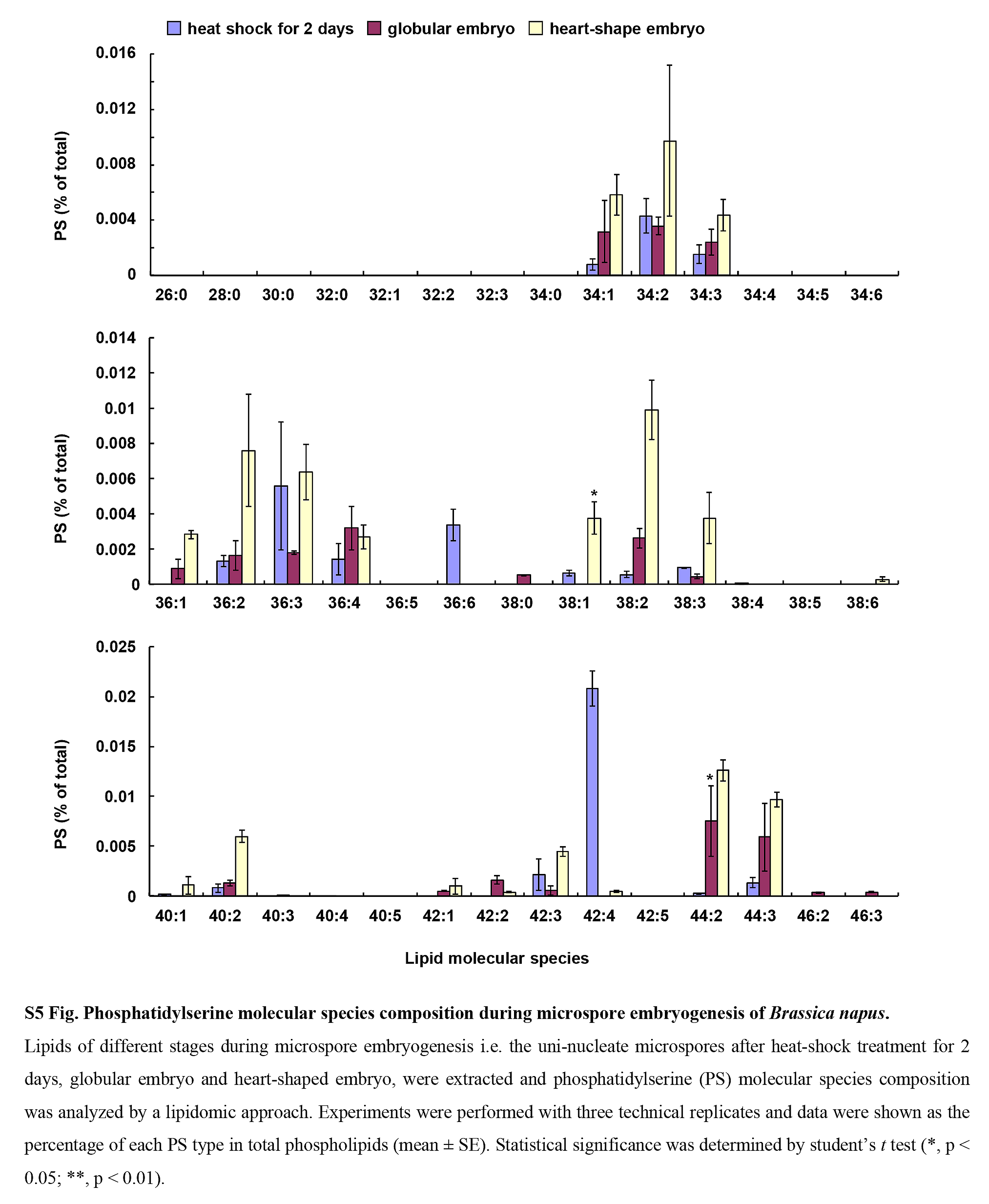

Supplement: S5 Fig — Lipids of different stages during microspore embryogenesis i.e. the uni-nucleate microspores after heat-shock treatment for 2 days, globular embryo and heart-shaped embryo, were extracted and phosphatidylserine (PS) molecular species composition was analyzed by a lipidomic approach. Experiments were performed with three technical replicates and data were shown as the percentage of each PS type in total phospholipids (mean ± SE). Statistical significance was determined by student’s t test (*, p < 0.05; **, p < 0.01). (TIF) [file pgen.1010320.s005.tif]

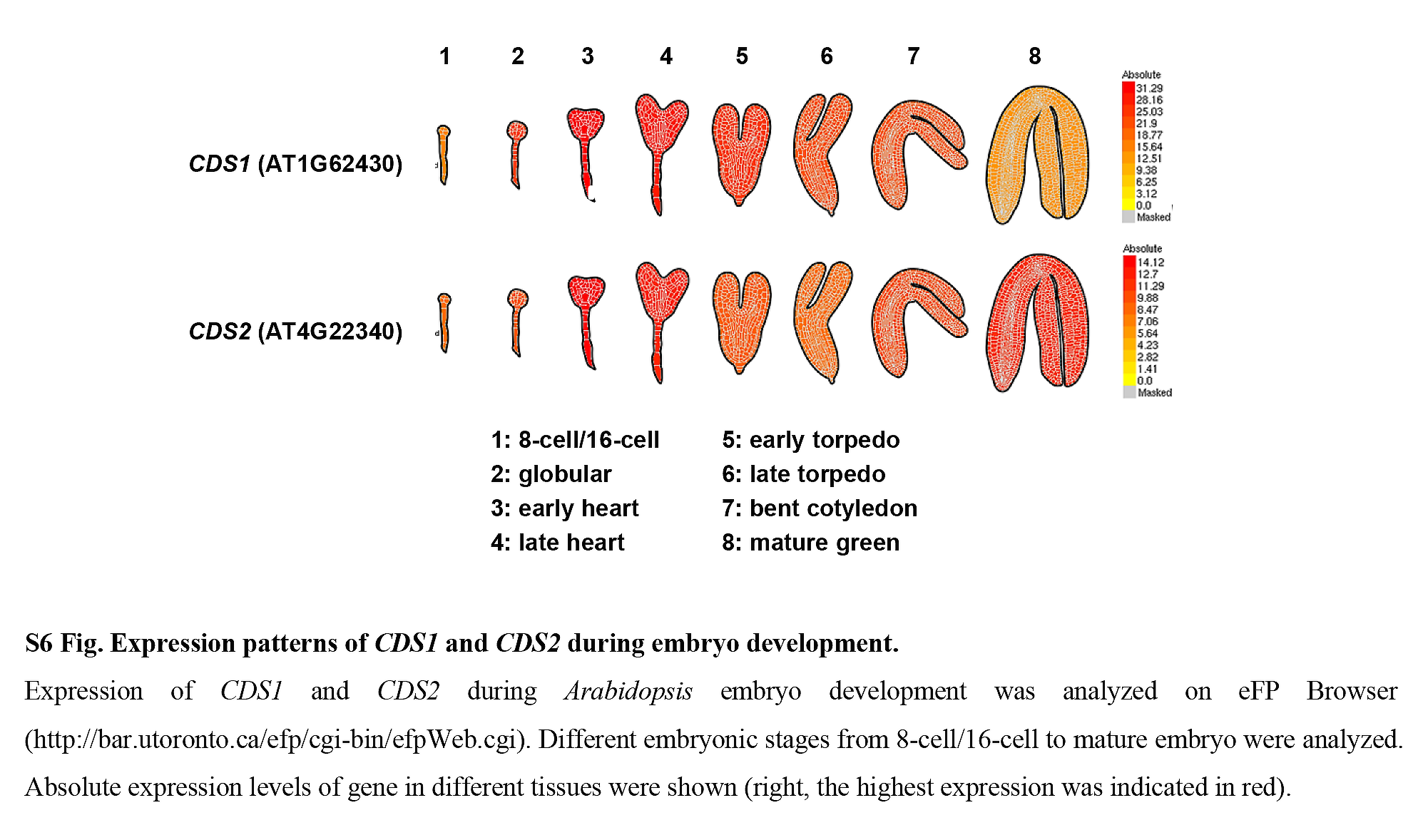

Supplement: S6 Fig — Expression of CDS1 and CDS2 during Arabidopsis embryo development was analyzed on eFP Browser (http://bar.utoronto.ca/efp/cgi-bin/efpWeb.cgi). Different embryonic stages from 8-cell/16-cell to mature embryo were analyzed. Absolute expression levels of gene in different tissues were shown (right, the highest expression was indicated in red). (TIF) [file pgen.1010320.s006.tif]

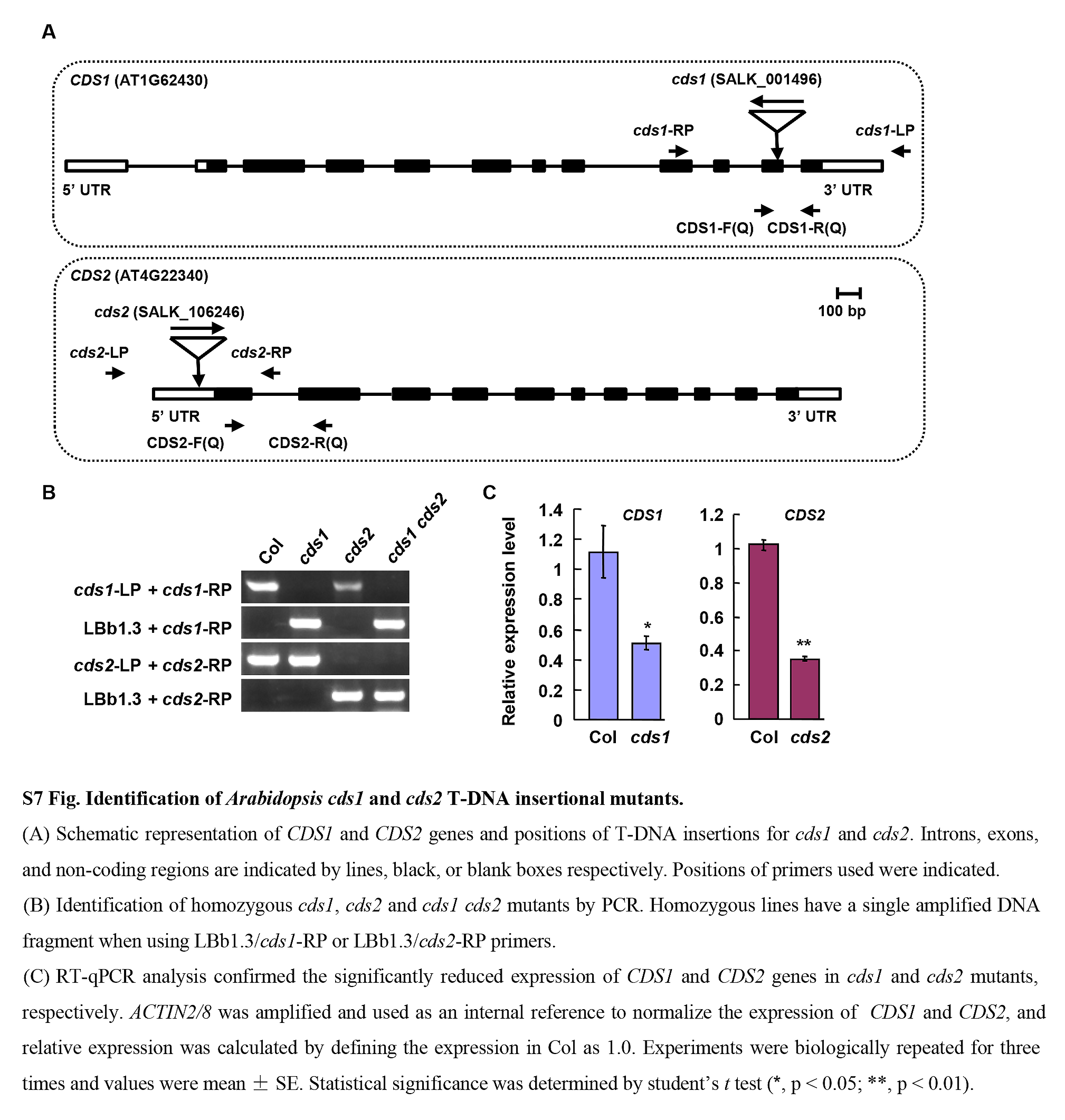

Supplement: S7 Fig — (A) Schematic representation of CDS1 and CDS2 genes and positions of T-DNA insertions for cds1 and cds2. Introns, exons, and non-coding regions are indicated by lines, black, or blank boxes respectively. Positions of primers used were indicated. (B) Identification of homozygous cds1, cds2 and cds1 cds2 mutants by PCR. Homozygous lines have a single amplified DNA fragment when using LBb1.3/cds1-RP or LBb1.3/cds2-RP primers. (C) RT-qPCR analysis confirmed the significantly reduced expression of CDS1 and CDS2 genes in cds1 and cds2 mutants, respectively. ACTIN2/8 was amplified and used as an internal reference to normalize the expression of CDS1 and CDS2, and relative expression was calculated by defining the expression in Col as 1.0. Experiments were biologically repeated for three times and values were mean ± SE. Statistical significance was determined by student’s t test (*, p < 0.05; **, p < 0.01). (TIF) [file pgen.1010320.s007.tif]
