# Supplementary material for: Cytidinediphosphate diacylglycerol synthase—Mediated phosphatidic acid metabolism is crucial for early embryonic development of Arabidopsis
Source: PLoS Genet. 2022 Jul 25;18(7):e1010320. doi: 10.1371/journal.pgen.1010320 (PMC9352201; doi:10.1371/journal.pgen.1010320)
Supplement: S2 Table — Added restriction enzyme site was underlined. (DOCX) [file pgen.1010320.s009.docx]

**Supplemental Table 2. Sequences of primers used in this study.** Added restriction enzyme site was underlined.

| **Primer name** | **Sequence (5’ to 3’)** |  |
| --- | --- | --- |
| **Primers for mutants identification** | | |
| *cds1*-LP | TGGTCTTTGGTGCGATAATTC | *cds1* T-DNA mutant identification |
| *cds1*-RP | GCACCTTTTGGAGGATTTTTC |  |
| *cds2*-LP | TCAACTCGATTTCTCTGCTCC | *cds2* T-DNA mutant identification |
| *cds2*-RP | CCAAAAACAATGTGAATTCCG |  |
| LBb1.3 | ATTTTGCCGATTTCGGAAC | T-DNA identification |
| **Primers for constructs** | | |
| pCDS1-GUS-F  (Hind III) | CGACGGCCAGTGCCAAGCTTGATGTTTGTTTCCCTTTCATTTCG | pCDS1:GUS |
| pCDS1-GUS-R  (Sal I) | GGGGATCCTCTAGAGTCGACAGCTCTTAAAAATTTGATTTTGCAG |  |
| pCDS2-GUS-F  (Hind III) | CGACGGCCAGTGCCAAGCTTAGCCCTGGAAAGAAACTGAC | pCDS2:GUS |
| pCDS2-GUS-R  (Sal I) | GGGGATCCTCTAGAGTCGACCACCTTAGTCTAACGTCAGAATACA |  |
| pCDS2-CDS2-F  (BamH I) | AGCTCGGTACCCGGGGATCCTCCTCGTTACTTGATGTTCC | pCDS2:CDS2 |
| pCDS2-CDS2-R  (Hind III) | CGACGGCCAGTGCCAAGCTTTTGTAAGAGAATTGTAGTGAGCT |  |
| **Primers for RT-qPCR** | | |
| CDS1-F | TTGTCTCCCAAAGCGTTTCG |  |
| CDS1-R | TGAGAGCTTGTCCTTCAGCA |  |
| CDS2-F | AAATTGCTGGTGATGCTCCA |  |
| CDS2-R | CCACCATGGCTGTAATGTAGAG |  |
| ACTIN2/8-F | ACGGTAACATTGTGCTCAGTGGTG |  |
| ACTIN2/8-R | CTTGGAGATCCACATCTGCTGGA |  |
